# Motivations to engage in collective action: A latent profile analysis of refugee supporters

**DOI:** 10.1111/bjso.12786

**Published:** 2024-07-09

**Authors:** Lisette Yip, Emma F. Thomas, Ana‐Maria Bliuc, Mihaela Boza, Anna Kende, Morgana Lizzio‐Wilson, Gerhard Reese, Laura G. E. Smith

**Affiliations:** ^1^ Flinders University Adelaide South Australia Australia; ^2^ University of Dundee Dundee UK; ^3^ Alexandru Ioan Cuza University Iași Romania; ^4^ Eötvös Loránd University Budapest Hungary; ^5^ University of Exeter Exeter UK; ^6^ University of Kaiserslautern‐Landau Kaiserslautern and Landau Germany; ^7^ University of Bath Bath UK

**Keywords:** collective action, latent profile analysis, performative allyship, refugees, self‐determination theory, social identity approach

## Abstract

What motivates people to participate in collective action? Some actions such as symbolic or online actions are often critiqued as performative allyship, motivated by personal gain rather than genuine concern for the cause. We aim to adjudicate this argument by examining the quality of motivations for acting, drawing on the insights of self‐determination theory and the social identity approach. Using latent profile analysis, we examined whether there are different types of supporters of refugees based on their underlying motives. In Study 1, we surveyed supporters of Syrian refugees from six nations (*N* = 936) and measured autonomous and controlled motivation, pro‐refugee identification and collective action. In Study 2 (*N* = 1994), we surveyed supporters of Ukrainian refugees in Romania, Hungary and the UK. We found 4–5 profiles in each sample and consistently found that supporters with high autonomous motivation take more action than disengaged or ambivalent supporters (low/neutral on all motives). However, contrary to the tenets of self‐determination theory, those high in both autonomous and controlled motives were the most engaged. We conclude that the most committed supporters are those with multiple motives, but further research is needed on the role of controlled motivation.

## BACKGROUND

In September of 2015, a photograph of a Syrian refugee toddler named Aylan Kurdi went viral after his body was found on a beach in Turkey while his family was fleeing conflict in Syria. The photo captured the world's attention and was shared widely on social media, garnering over 30,000 tweets in the first 12 h after its publication (Vis & Goriunova, 2015). Several years later, Russia launched an invasion of Ukraine, resulting in nearly six million refugees fleeing Ukraine by the end of 2022 (UNHCR, 2023). These events also sparked an international wave of public displays of support for refugees, with #IStandWithUkraine trending online, Ukrainian flags displayed in homes and businesses, on bumper stickers, social media profiles and more.

Posting on social media, displaying stickers and flags are all examples of what is sometimes critiqued as performative allyship (Kutlaca & Radke, 2023) – that is, low‐cost, low‐effort or symbolic actions that require little commitment from the actor. *Performative allyship* refers to solidarity‐based actions taken by advantaged group members ostensibly out of concern for a disadvantaged group (such as refugees) but actually for self‐serving reasons. For example, advantaged group members may want to feel good about themselves (known colloquially as slacktivism; Skoric, 2012) or portray themselves as moral and enhance their reputation (known as virtue‐signalling; Westra, 2021). This may at least partially explain why, despite the initial surge of support for Syrian refugees, the movement had lost momentum a year later and did not achieve its desired social change of resettling tens of thousands more refugees (Thomas et al., 2018).

However, other research highlights the importance of solidarity‐based actions, taken by advantaged group members, in achieving greater justice for disadvantaged groups (Subašić et al., 2008). Advantaged group members' denouncement of injustice is often taken more seriously (Drury & Kaiser, 2014; Gulker et al., 2013) and they can effectively encourage others to support a movement and can thus play a key role in contributing to social change (Kutlaca et al., 2022). Importantly, the critique of performative allyship focuses on the motivation behind the actions of allies and suggests that negative outcomes only occur when allies have self‐serving motives. For example, Cornelissen et al. (2013) found that expressing symbolic support for a cause undermined people's willingness to take further, more meaningful action, but only if they were motivated by impression management. Understanding the motivations underlying solidarity‐based actions may be key to understanding when allies take effective, meaningful action in support of disadvantaged groups, versus when action is fleeting, symbolic, or otherwise serves the needs of allies.

In this research, we examine whether there are distinctive subgroups of supporters that differ meaningfully based on their underlying motivations (see also Radke et al., 2020). For example, people may support refugees because it aligns with their beliefs and identity, or because it makes them feel like a good person, or both may be true. We anticipated that supporters driven by internalised motives would exhibit stronger commitment to (i.e., collective action and identification with) the cause, relative to those motivated by more external factors such as guilt or reputation management. However, some supporters may be driven by multiple motivations simultaneously and by different combinations of motives. Accordingly, we adopt a person‐centred approach (using latent profile analysis (LPA)) to understand how different combinations of motives predict the extent of a supporter's commitment to action. We do so in the context of support for refugees fleeing the conflict in Syria in 2015 (Study 1) and Ukraine in 2022 (Study 2).

### Understanding types of motivation using self‐determination theory

We draw on the insights of self‐determination theory, which provides a differentiated and fine‐grained analysis of motivation that may help to understand variation in why people engage with social movements like those to support refugees (Deci & Ryan, 1985; Ryan & Deci, 2000; see Table 1). Within this theoretical perspective, the focus is not on the strength of motivation, but on the quality or nature of the motivation. Broadly, different types of motives fall into two categories. *Autonomous motivation* underlies behaviour that works towards a genuinely desired outcome and expresses the individual's identity and interests (Weinstein & Ryan, 2010). In contrast, *controlled motivation* refers to external pressures that drive behaviour, meaning it is less self‐determined or personally valued (Sheldon & Elliot, 1998).

**TABLE 1:** The subtypes of motivation outlined in self‐determination theory (Deci & Ryan, 1985).

	Non‐regulation	Controlled motivation		Autonomous motivation		
	Amotivation	External regulation	Introjected regulation	Identified regulation	Integrated regulation	Intrinsic motivation
	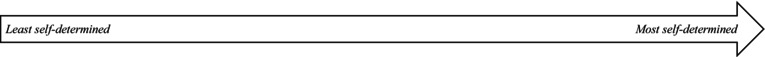					
Definition	‘Going through the motions’ without valuing the behaviour or expecting to achieve any desired outcomes	Behaviour is performed for reasons external to the self and is motivated by reward or punishment	External regulations are ‘taken in’ but not personally valued; behaviour is motivated by shame, guilt, anxiety, pride, or self‐esteem	Behaviour is personally valued and accepted as true to the self as its outcomes are consistent with one's goals	Behaviour is highly meaningful and associated with one's deeply held values; it is assimilated with other aspects of self	Behaviour is performed for the enjoyment of the activity with no focus on future goals or outcomes
Example	‘I have no reason to participate in actions to support refugees. I don't think it will achieve anything’	‘I participate in actions to support refugees because my friends will criticise me if I don't’	‘I participate in actions to support refugees because I would feel bad if I didn't’	‘I participate in actions to support refugees because I want to improve conditions for refugees’	‘I participate in actions to support refugees because my passion for human rights is a core part of who I am’	‘I participate in actions to support refugees because it is satisfying and rewarding’

These broad categories can be broken down into more fine‐grained subtypes of motivation existing on a continuum of self‐determination. Autonomous motivation is comprised of three subtypes: *intrinsic motivation*, the most self‐determined form of motivation which refers to enjoyment and interest in the activity itself (Burton et al., 2006), *integrated regulation*, when behaviour is highly meaningful and integrated with one's deeper sense of self and *identified regulation*, when behaviour is personally important and consistent with one's goals, but not assimilated with other aspects of self (Ferguson et al., 2015). On the other hand, controlled motivation is comprised of two subtypes; *introjected regulation* involves attaining positive self‐evaluations (e.g., feeling pride) or avoiding negative feelings such as shame or guilt (Koestner et al., 1996), while *external regulation* is the motive to gain rewards or avoid punishments (Deci et al., 1999).

Autonomous motivation has been found to predict greater engagement, performance and persistence than controlled motivation in a range of contexts. People whose behaviour is more autonomous are less likely to drop out of high school (Vallerand et al., 1997) and more likely to engage in effective healthcare (Williams et al., 2004), persist at their chosen sport (Pelletier et al., 2001) and vote in elections (Koestner et al., 1996). However, there is minimal work examining these forms of motivation to engage in collective action (but see related approaches on outgroup discrimination; Amiot et al., 2012, 2014; support for charitable causes; Ferguson et al., 2015; volunteering; Geiser et al., 2014). While self‐determination theory traditionally examines how autonomous (vs. controlled) motivation drives behaviour towards personal goals (e.g., sporting achievements, education), we extend this to consider the role of motivation in pursuit of group goals (Amiot et al., 2020; Thomas et al., 2017).

### Outcomes of autonomous and controlled motivation: opinion‐based group identification and collective action

In the present research, we seek to overcome reductionist debates about whether support is driven by self‐ or other‐interested motives to distinguish between supporters of refugees who are driven by autonomous or controlled motivation, or combinations of both. We propose that these motives can predict the likelihood and frequency of supporters engaging in collective action and the degree of identification with supporters of refugees (see also Yip et al., 2024). Self‐determination theory suggests that autonomously motivated behaviour reflects one's authentic self, identity and interests. In the context of collective action where a group‐level self is a key predictor (Agostini & van Zomeren, 2021), autonomously motivated behaviour may be true to one's *social identity* and reflect one's deeply held, internalised commitment to the group (Turner et al., 1987). Thus, internalised motivation towards collective goals is closely linked with social identification (Yip et al., 2024). We therefore propose that the underlying motivation will be associated with variation in opinion‐based group identification as a supporter of refugees. Opinion‐based group identities form on the basis that a stance on an issue is a core part of ‘who I am,’ and this internalised sense of agreement is shared among the group (Thomas et al., 2016). Thus, these identities should be preceded by autonomous motivation; the more one is autonomously motivated to support refugees, the more likely they are to identify with a group whose norms and ethos is based on a shared support for refugees. In contrast, those who act for more external reasons are expected to report lower levels of internalised identification.

Autonomous motivation is associated with greater engagement in individual behaviour such as pursuing education or sport; we expect that this finding will extend to collective action and that people driven by autonomous motives to support a particular cause will take more action than those with controlled motives (Yip et al., 2024). Furthermore, the association between motivation and identification can also explain why some supporters are more committed to action than others, as opinion‐based group identification is a well‐established predictor of collective action (Agostini & van Zomeren, 2021; Bliuc et al., 2007; Thomas et al., 2022). Autonomously motivated supporters will identify more strongly with the group and therefore take more action; in contrast, controlled motives have been shown to undermine group identification and, in turn, collective action (Yip et al., 2024).

### Using a person‐centred approach to adjudicate the presence and effects of mixed motives

Some supporters may be driven by multiple motives simultaneously. That is, within people (and beyond the independent effects of autonomous and controlled motivation), some supporters may be high in both autonomous and controlled forms of motivation. Person‐centred statistical approaches in self‐determination theory have previously identified subgroups of people high in both autonomous and controlled motivation (e.g., volunteering; Geiser et al., 2014; workplace engagement; Levesque‐Côté et al., 2021) where mixed motives predict moderate levels of engagement relative to people motivated purely by autonomous (high engagement) or controlled motives (low engagement). We may similarly find that some supporters of refugees are driven by multiple motives and expect that such supporters will evidence moderate levels of engagement.

However, self‐determination theory contends that controlled and autonomous motives lie on opposite ends of a continuum (Ryan & Deci, 2000) and should not co‐occur, as influences that foster controlled motivation can crowd out autonomous motivation (Gagné & Forest, 2008). For example, in the context of refugee supporters, wanting to appear moral should reduce autonomous motivation as it shifts the focus to reputation management and away from the plight of refugees. As a result, controlled motivation would increase, but autonomous motivation – and, in turn, collective action – would decrease. A person‐centred approach will help adjudicate whether individuals can hold mixed motives for supporting refugees and whether it is the quality of motivation that matters for collective action (i.e., autonomous motivation alone is better than multiple motives) or quantity (multiple motives lead to more action).

### Motivation develops within the social and cultural context

Self‐determination theory contends that motivation is context‐specific and is influenced by factors within the individual's environment. For example, school students with autonomy‐supportive teachers are more likely to feel autonomously motivated to complete schoolwork, compared to those with coercive or controlling teachers (Ryan & Deci, 2000). We expect that context will similarly play a role in the motives that individuals develop to act in support of refugees. For example, collective action is more normative in some countries and cultural attitudes towards refugees vary. Social movements and their supporters are a product of the social and political contexts in which they form (Thomas et al., 2022; Uluğ et al., 2022) and the motivational quality of subgroups of supporters may differ across distinct socio‐political contexts. Thus, in Study 1 we sample from multiple nations and in Study 2 we examine cross‐national differences in the motives of refugee supporters.

### The current research

We tested the proposition that the underlying motivation for taking collective action differs among supporters of refugees. We conducted a latent profile analysis (LPA), a method used to identify subgroups of people based on shared characteristics that are different from other subgroups (Osborne & Sibley, 2017). LPA focuses on characteristics of people, rather than testing relationships between variables and can identify subgroups of refugee supporters who meaningfully differ based on their underlying motivations (Thomas & McGarty, 2018). The advantage of this methodology is that it allows for the examination of diverse reactions to social issues and patterns of action engagement among supporters of a social movement (see, for example, Álvarez et al., 2024; Lizzio‐Wilson et al., 2021) and does not assume that a single population is homogeneous. For detailed discussion on the use of LPA in collective action research, see Thomas et al. (In press).

**FIGURE 1:**
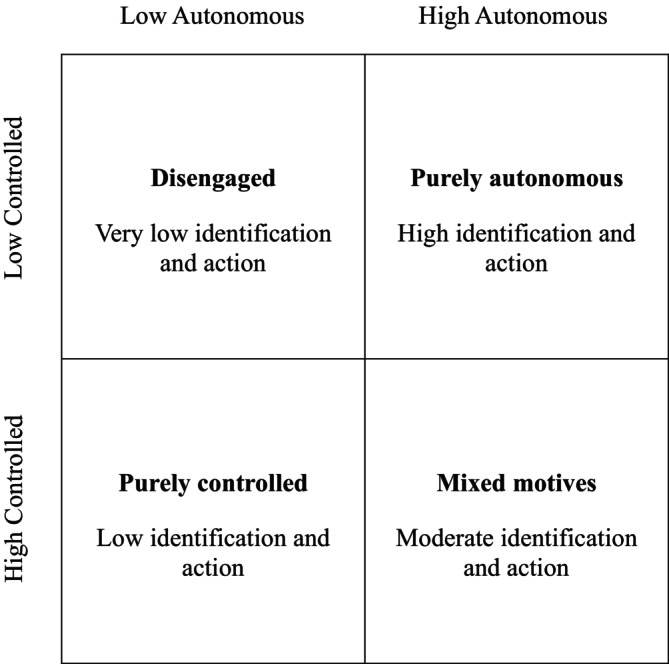
Four potential combinations of autonomous and controlled motivations and their hypothesised outcomes.

It is important to note that LPA is inherently exploratory and inductive. The analysis is repeated with increasing numbers of profiles and the optimal solution is determined based on statistical and theoretical fit (criteria described below). We did not have specific a priori expectations about the number and nature of profiles. Nevertheless, our approach was guided by theoretically derived expectations about the likely outcomes of (combinations of) autonomous and controlled motivation (see Figure 1). We expected that membership of profiles higher in autonomous motivation (i.e., those defined by higher levels of identified and/or integrated regulation) would predict greater collective action and identification as a supporter of refugees than other profiles (H1). In contrast, membership of profiles higher in controlled motivation (i.e., those defined by higher levels of external and/or introjected regulation) would predict lower levels of action and identification than other profiles (H2). Profiles low in both types of motivation would predict the lowest levels of action and identification (H3). Profiles high in both types of motivation would predict moderate levels of action and identification (H4).

### Openness and transparency

The hypotheses and analyses for Study 1 were pre‐registered after data collection but prior to conducting analyses; see https://osf.io/fkp4t/?view_only=b6dc9eb0f59c4bf8a65dec0e8c3e3dc0. To minimise the researcher degrees of freedom, we pre‐registered that we would test for up to seven profiles, as the literature on self‐determination theory tends to find a maximum of six (e.g., Geiser et al., 2014; Gillet et al., 2017). The process of assessing the number of profiles (*k*) with the best fit involves comparing the *k* − 1 solutions. Therefore, we pre‐registered testing up to seven so that we could empirically assess the 6‐profile solution. We also pre‐registered analyses testing for other predictors of profile membership, described in the Supporting Information in the interests of scope (for Study 1 and 2). The numbering of hypotheses here relative to the pre‐registration therefore differs slightly. Finally, we conducted an exploratory analysis testing whether profile membership predicted engagement in specific actions, to test whether some profiles might be associated with taking more public and low‐effort (i.e., potentially performative or slacktivist) actions relative to others, but do not report this analysis here as the findings were similar across different types of actions (see the Supporting Information for details).

## STUDY 1

Data were collected in September and October 2015, at the height of the public response to Syrian refugees following the dissemination of the image of Aylan Kurdi. We surveyed participants in six countries who indicated that they support global action to support Syrian refugees. We only included supporters as we were interested in understanding motivations for acting to support refugees; such measures would not be meaningful for non‐supporters. The identification and collective action measures were previously reported by Thomas et al. (2019) however the focal measures of motivation have not been published nor have the data been analysed in this way. We used integrated, identified, introjected and external regulation as indicators to determine the profile structure. We used these subtypes of motivation rather than aggregate measures of autonomous and controlled motivation to provide a more fine‐grained analysis; LPA can determine whether such a distinction is necessary as it will identify whether subtypes co‐occur in the profiles. We tested how profile membership predicted opinion‐based group identification as a supporter of refugees and collective action using three measures: action intentions, self‐reported past action and a behavioural measure of donation.

### Methods

#### Participants

All participants gave informed consent before beginning the survey. We removed participants who indicated that they were not supporters of global action to support Syrian refugees and were left with 955 participants, of whom 19 had data missing on all indicators. The final sample included 936 supporters recruited from Hungary (*N* = 100), Germany (*N* = 154), the UK (*N* = 144), the US (*N* = 201), Australia (*N* = 284) and Romania (*N* = 72). The sample was 59.1% female with an average age of 31.41 (*SD* = 16.17). The original sample including both supporters and non‐supporters comprised of 65% students and 35% community members. However, we did not record student status in our study, so we do not know the exact proportion after removing non‐supporters from the analysis.

Sample size calculations are not straightforward for LPA. Power is determined by the number of indicators, participants and profiles and the degree of separation between profiles, which are all difficult to predict a priori (Tein et al., 2013). However, simulation studies suggest that a minimum *N* = 500 should provide sufficient power to accurately detect the number of latent profiles in a sample (Nylund et al., 2007; Spurk et al., 2020). The sample size was determined by practical constraints on data collection meaning we could not run separate analyses for each country, but the combined sample size should be sufficient to test the proposed profile solution.

#### Procedure

Students were recruited on university campuses or for course credit in all nations. In Romania, Germany, the US and Australia, community members were also recruited via public forums (e.g., Craigslist) and personal networks. The questionnaire titled, ‘Attitudes about the Syrian refugee crisis’ was distributed online via Qualtrics, or via hard copy. Responses were given on a Likert scale ranging from 1 (strongly disagree) to 7 (strongly agree), with scale items averaged to create a composite score, unless indicated otherwise below. The full questionnaire is available at https://osf.io/un4a5/?view_only=c0e007d9531043a9817808d0ae9a7fc9.

#### Materials

##### Motivation

Participants were asked to what extent they supported Syrian refugees because of two types of controlled motivation; external regulation (‘Because others would get mad at me if I didn't’); introjected regulation (‘Because I would feel like a bad person if I didn't’); and two types of autonomous motivation; identified regulation (‘Because I thought it was important to act in this way’); and integrated regulation (‘Because I valued doing so’). These were single‐item measures adapted from Weinstein and Ryan (2010).

##### Opinion‐based group identification

Three items adapted from Cameron (2004) measured opinion‐based group identification, e.g., ‘I identify with other supporters of Syrian refugees’ (*α* = .75).

##### Collective action intentions

Participants were asked to what extent they intended to take the following actions to support Syrian refugees: signing a petition, donating money, posting on social media and volunteering (*α* = .73).

##### Past collective action

Participants were asked if they had *already* taken the actions listed above and responded ‘yes’ or ‘no’ to each item. The number of ‘yes’ responses was summed to create a score ranging from 0 to 4.

##### Donation

At the conclusion of the survey, participants were informed that researchers would make a donation of 1USD on their behalf and were asked what proportion they would like to donate to Syrian refugees, or disadvantaged children in their own country. This was a continuous measure where participants could choose what proportion of the $1 to donate to each cause, in increments of 10c (e.g., 60c to Syrian refugees, 40c to children in their own country), coded as 1 (0c to Syrian refugees)‐11 ($1 to Syrian refugees). The researchers made the donations to each charity in line with participants' allocations (see Lizzio‐Wilson et al., 2022 for a similar methodology).

#### Analytic strategy

We conducted a LPA in Mplus version 8.6 (Muthén & Muthén, 1998–2017), using the four motivation items (integrated, identified, introjected and external regulation) as indicators to determine the profiles. We could not run a multi‐group LPA or conduct tests of invariance between countries, as this would require sufficient sample sizes to first test the model separately in each country (at least 500 participants). Best fit was determined by a smaller value on the Akaike's Information Criterion (AIC), Bayesian information criterion (BIC) and adjusted BIC and a significant (*p* < .05) Vuong–Lo–Mendell–Rubin test (VLMR) and bootstrapped likelihood ratio test (BLRT) indicating that the model has better fit than the previous iteration with fewer profiles (Asparouhov & Muthén, 2012; Tein et al., 2013). We also considered theoretical fit such that, for instance, the final solution should not have multiple profiles with little conceptual differentiation; this would be empirically supported by a higher entropy value suggesting greater differentiation between profiles.

### Results

We concluded that the five‐profile solution had the best fit with the data (Table 2). The five‐profile solution had a significant VLMR and lower AIC, BIC and aBIC than the four‐profile solution.

**TABLE 2:** Fit statistics for solutions with 1–7 profiles.

*k*	AIC	aBIC	BIC	VLMR, *p* value	BLRT, *p* value	Entropy	Size of profiles
1 profile	12,654.58	12,667.91	12,693.31	–	–	–	100%
2 profiles	12,269.57	12,291.22	12,332.51	<.001	<.001	0.896	85%/15%
3 profiles	11,921.79	11,951.77	12,008.94	<.001	<.001	0.909	77%/16%/7%
4 profiles	11,693.72	11,732.03	11,805.08	<.001	<.001	0.854	46%/30%/16%/7%
5 profiles	11,601.84	11,648.47	11,737.40	.028	<.001	0.860	46%/30%/10%/7%/7%
6 profiles	11,489.69	11,544.65	11,649.46	.160	<.001	0.822	41%/22%/16%/8%/7%/6%
7 profiles	11,387.93	11,451.22	11,571.91	.252	<.001	0.831	38%/24%/16%/8%/7%/5%/1%

**FIGURE 2:**
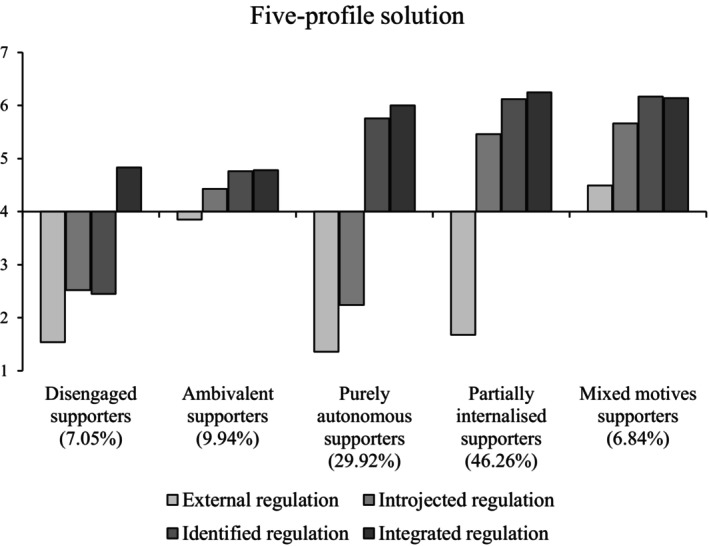
Profile means for each type of motivation in the five‐profile solution.

Indicator means for the five profiles are illustrated in Figure 2. First, *disengaged supporters* were low on external, introjected and identified regulation. Integrated regulation was somewhat higher, but we labelled these supporters as disengaged as they actively disagreed that most of the motives were applicable to them. Next, *ambivalent supporters* were near the scale midpoint for all four types of motivation. *Purely autonomous supporters* were high in autonomous motivation (identified and integrated regulation) and low in controlled motivation (external and introjected regulation). *Partially internalised supporters* were low in external regulation, but high in introjected, identified and integrated regulation. Finally, *mixed motives supporters* scored highly on all types of motivation, although relatively lower on external regulation than other motives.

After determining the optimal solution, we used the AUXILIARY option in Mplus to examine opinion‐based group identification, action intentions, past action and donation as outcomes of profile membership (Table 3). Disengaged and ambivalent supporters had the lowest levels of identification and all measures of action compared to all other groups, followed by purely autonomous supporters, then partially internalised supporters. Mixed motives supporters reported higher identification than purely autonomous and partially internalised supporters and greater action intentions than purely autonomous supporters. Past action and donation were not significantly different between mixed motives supporters compared to partially internalised and purely autonomous profiles.

**TABLE 3:** Means (SE) on the outcome measures for each latent profile.

	Disengaged	Ambivalent	Purely autonomous	Partially internalised	Mixed motives
Identification	4.45 (.13)_a_	4.74 (.09)_a_	5.44 (.05)_b_	5.88 (.04)_c_	6.15 (.10)_d_
Action intentions	3.58 (.15)_a_	3.86 (.12)_a_	4.66 (.07)_b_	5.20 (.06)_c_	5.00 (.15)_c_
Past action	0.57 (.11)_a_	0.45 (.08)_a_	0.99 (.07)_b_	1.38 (.06)_c_	1.12 (.15)_bc_
Donation	7.21 (.43)_a_	6.42 (.37)_a_	8.21 (.18)_b_	8.86 (.14)_c_	8.65 (.36)_bc_

*Note*: Subscripts denote where profiles are significantly different at *p* < .05.

### Discussion

We found partial support for our hypotheses. Profiles higher in autonomous motivation identified more strongly with refugees and took more action than those lower in autonomous motivation, consistent with H1. However, profiles high in controlled motivations were more engaged in action and identified more strongly than those low in controlled motivation, thus H2 was not supported. We also found support for H3, as the least engaged profiles of supporters were relatively low in all motives. Finally, H4 was not supported, as a combination of motives led to higher commitment to action than autonomous motives alone (contrary to Geiser et al., 2014). It therefore appears that autonomous motivation is good for collective action – but *also* having controlled motivation is better.

The findings also contradict the assertion that autonomous and controlled motives are opposite ends of a continuum and crowd each other out (Deci et al., 1999; Gagné & Forest, 2008). About half the sample scored highly on both types of autonomous motivation and at least one type of controlled motivation. However, most of these were in the partially internalised group, where introjected regulation was high and external regulation was low. While introjected regulation is categorised as a form of controlled motivation, it is considered to be partially internalised as it stems from one's own self‐evaluations and emotions (e.g., desire to feel pride, avoid guilt) and is not externally imposed (Ryan & Deci, 2017). In the context of collective action and motivations towards group‐level goals, introjected and autonomous motivations may be more strongly associated than in other contexts. This is because people who care about a cause may also feel guilt or shame if they do not meet their own internalised standards and those of the group with which they identify. External regulation and autonomous motivation may be similarly associated in the group context, as those who identify most strongly with the group are more likely to care what other members think and conform to norms and expectations of the group (Reynolds et al., 2015). Thus, the self‐determination continuum structure may be less rigid in the context of group goals, where meeting the expectations of others is not as externalised or peripheral as it may be in the individual context.

Also of note is that we did not identify a group exclusively high in controlled motivation. The analysis therefore suggests that there is no significant quantity of supporters driven solely, or even primarily, by controlled motivation (i.e., ‘slacktivists’ or ‘performative activists’). We conducted Study 2 in a different refugee context to determine whether these findings would be replicated, using more robust two‐item measures of each type of motivation and changing the wording for some items (see below).

Finally, although we had data from multiple nations in Study 1, we did not have sufficient sample sizes to detect different subgroups within each nation. We note that differences across nations, including both contextual differences and characteristics within our sample (e.g., different proportions of students) are a key limitation, as these factors are likely to exert significant influence over the types of supporters and their relative prevalence. Thus, the five profiles we identified do not reflect subgroups of one population, but rather generalisations across national contexts. Furthermore, due to the small sample sizes in some countries, our analysis would fail to detect any smaller subgroups that may be present only in a single country. Larger sample sizes would be required to obtain a more comprehensive picture of the subgroups of supporters that may be present in each country and whether these differ across national contexts. We also only had single‐item measures of motivation and did not have measures of intrinsic motivation or amotivation. We address these limitations in Study 2.

## STUDY 2

Study 2 was conducted in the context of refugees fleeing Ukraine following the Russian invasion beginning in February 2022. The invasion and resulting refugee crisis were ongoing throughout the study (December 2022–May 2023). We pre‐registered the analyses for Study 2 prior to data collection; see https://osf.io/frdzm/?view_only=50535ff8d5d94b5cae5f2c2fe362b529.

We expected that profiles higher in autonomous motivation would score higher on all outcomes than those lower in autonomous motivation (H1). Based on the findings of Study 1 but contrary to our original hypotheses, we expected that profiles relatively high in *both* autonomous and controlled motivation would have the highest scores on all outcomes relative to all other profiles (H2). We did not expect to find profiles where controlled motivation is high, but autonomous motivation is low (H3).

Study 2 also included measures of amotivation and intrinsic motivation. Amotivation refers to an absence of motivation, when one does not expect to achieve any desired outcomes from the behaviour (Table 1). We expected that profiles higher in amotivation would be relatively low on all other indicators (H4) and thus would score lowest on all outcomes relative to other profiles (H5).

Intrinsic motivation is considered the most internalised form of motivation, when the behaviour is experienced as inherently enjoyable (Ryan & Deci, 2000). We expected that profiles high in other forms of autonomous motivation would also be high in intrinsic motivation (H6a). These motives are adjacent on the self‐determination continuum and thus should be highly correlated (Howard et al., 2017). However, we expected that motives derived from one's sense of self and personal goals (i.e., integrated and identified regulation), rather than enjoyment, would be more strongly predictive of collective action (H6b). Research on voting behaviour has found that identified and integrated regulation are associated with voting, but intrinsic motivation is not (Koestner et al., 1996); intrinsic motivation may be less relevant for collective action, which is necessary for social change (Thomas & Louis, 2013) but unlikely to be experienced as inherently enjoyable.

We also considered potential cross‐national differences between supporters of refugees due to differences in the socio‐political context. We recruited at least 500 participants from three different nations, allowing us to run separate analyses for each country and conduct cross‐national comparisons via a multi‐group LPA. We did not pre‐register hypotheses regarding the nature of differences between countries but sought to establish whether similar profiles would be present on an exploratory basis.

### Methods

#### Participants

We pre‐registered that we would collect data in Romania, Hungary and Germany, but instead sampled UK participants due to difficulties collecting the German data. We recruited 1994 participants (Romania *N* = 736, Hungary *N* = 756, the UK *N* = 502). Mean age varied across samples (Romania = 32.45, *SD* = 13.04, Hungary = 27.24, *SD* = 12.20, the UK = 42.29, *SD* = 13.58) and more than half of participants were female (Romania = 51.6%, Hungary = 72.8% and the UK = 57.2%).

#### Procedure

In Hungary, data were collected between December 2022–March 2023. Students (78% of the sample) were recruited for course credit, supplemented by a community sample who were offered a raffle prize (one of three €30 gift cards). In Romania, data were collected in January‐February 2023. Students (approximately 27% of the sample) were offered additional course credits for recruiting friends and family to complete the survey. In the UK, we administered the survey in May 2023 to a community sample via Prolific Panels and paid participants £2 for completing the questionnaire. The questionnaire titled, ‘Attitudes towards Ukrainian refugees’ was translated from English to Romanian and Hungarian and distributed online via Qualtrics. As with Study 1, we only recruited people who indicated that they support global action in support of Ukrainian refugees. Responses were given on a scale from 1 (strongly disagree) to 7 (strongly agree) unless indicated otherwise.

#### Materials

##### Motivation

Participants were asked to indicate why they take action to support Ukrainian refugees. There were two items for each type of regulation measured in Study 1; integrated regulation (*r* = .70–.76), identified regulation (*r* = .63–.83), introjected regulation (*r* = .31–.41) and external regulation (*r* = .42–.68). We added two‐item measures of amotivation (e.g., ‘I don't know, I don't really have any good reason to do so’; *r* = .48–.71) and intrinsic motivation (e.g., ‘Because I experience satisfaction from engaging in this behaviour’; *r* = .62–.80). The two items were averaged to create a composite score of each motivation. Most of the items from Study 1 were retained, but some were revised slightly, e.g., ‘Because others would get mad at me if I didn't’ was changed to ‘Because others would think badly of me if I didn't’ as people may be more likely to fear judgement from peers than outright anger. The revised measure may therefore be more likely to detect external regulation within the sample.

##### Opinion‐based group identification

The same items were used as in Study 1 (*α* = .87–.90).

##### Collective action intentions

Participants were asked to what extent they intended to take the following actions to support Ukrainian refugees: posting on social media, carrying or displaying an item to show support, updating social media profile to show support, signing a petition, talking to friends, family or colleagues, writing an email to a politician, donating, attending a rally, volunteering and offering a place to stay in their home (*α* = .90–.91).

##### Past collective action

Participants were asked if they had *already* done any of the above actions and responded ‘yes’ or ‘no’ to each item. The number of ‘yes’ responses was summed, ranging from 0 to 10.

##### Donation behavioural measure

At the conclusion of the survey, we provided a link to a webpage where participants could donate to assist Ukrainian refugees. However, few participants clicked on this link and there were no significant differences in clicks between any profiles, so this will not be discussed further.

#### Analytic strategy

As we had more than 500 participants in each sample, we conducted a multi‐group LPA. To run a multi‐group LPA, it is first necessary to conduct separate LPAs using each sample (Morin et al., 2016). If the same number of profiles is identified in the optimal solution for each sample (thus establishing configural similarity), it is then possible to combine samples and test for structural, dispersion and distributional similarity across countries (described in more detail below). Thus, we first describe the profiles in each sample, then discuss the multi‐group analysis.

### Results

We used six motivation items (amotivation, external regulation, introjected regulation, identified regulation, integrated regulation and intrinsic motivation) as indicators to determine the profiles. The fit statistics for solutions with 1–7 profiles are displayed in Table 4. We modelled collective action intentions, past action and opinion‐based group identification as outcomes (Table 5).

**TABLE 4:** Fit statistics for each solution up to seven profiles.

*k*	AIC	aBIC	BIC	VLMR, *p* value	BLRT, *p* value	Entropy	Size of profiles
Romania							
1 profile	15,322.524	15,339.635	15,377.739	–	–	–	100%
2 profiles	14,048.248	14,075.340	14,135.671	<.001	<.001	0.883	69%/31%
3 profiles	13,479.372	13,516.445	13,599.004	<.001	<.001	0.912	60%/35%/6%
4 profiles	13,215.935	13,262.989	13,367.775	.002	<.001	0.894	52%/34%/8 %/6%
5 profiles	13,008.570	13,065.606	13,192.620	.033	<.001	0.845	39%/26%/20%/9%/6%
6 profiles	12,893.227	12,960.243	13,109.484	.302	<.001	0.833	29%/21%/20%/18%/6%/6%
7 profiles	12,787.128	12,864.126	13,035.595	.007	<.001	0.821	21%/20%/17%/17%/13%/6%/6%
Hungary							
1 profile	15,563.365	15,580.797	15,618.902	–	–	–	100%
2 profiles	14,506.154	14,533.754	14,594.087	.001	<.001	0.845	73%/27%
3 profiles	13,832.342	13,870.110	13,952.671	<.001	<.001	0.879	51%/42%/7%
4 profiles	13,585.732	13,633.669	13,738.458	.024	<.001	0.845	42%/36%/17%/5%
5 profiles	13,420.347	13,478.452	13,605.469	.047	<.001	0.813	33%/25%/19%/18%/5%
6 profiles	13,277.449	13,345.723	13,494.967	.028	<.001	0.843	38%/26%/17%/10%/5%/5%
7 profiles	13,175.542	13,253.984	13,425.456	.150	<.001	0.850	36%/25%/17%/10%/5%/5%/1%
The UK							
1 profile	9623.517	9636.052	9674.140	–	–	–	100%
2 profiles	9072.413	9092.259	9152.567	.007	<.001	0.804	67%/33%
3 profiles	8818.720	8845.878	8928.404	.009	<.001	0.885	60%/38%/3%
4 profiles	8599.614	8634.083	8738.828	.019	<.001	0.879	51%/35%/10%/5%
5 profiles	8486.462	8528.243	8655.206	.155	<.001	0.828	36 %/31%/18%/10%/5%
6 profiles	8408.088	8457.180	8606.362	.231	<.001	0.817	31%/30%/15%/14%/8%/2%
7 profiles	8323.421	8379.825	8551.226	.539	<.001	0.820	24%/23%/23%/20%/5%/3%/2%

**TABLE 5:** Means (SE) on the outcome measures for each latent profile.

Romania	Disengaged	Ambivalent	Partially internalised	Mixed motives
Identification	3.75 (.20)_a_	4.90 (.07)_b_	6.01 (.04)_c_	6.23 (.08)_d_
Action intentions	2.05 (.11)_a_	3.55 (.06)_b_	4.80 (.05)_c_	5.74 (.11)_d_
Past action	0.54 (.11)_a_	2.01 (.10)_b_	4.17 (.11)_c_	4.90 (.28)_d_

Hungary	Disengaged	Ambivalent	Partially ambivalent	Partially internalised
Identification	3.24 (.21)_a_	4.51 (.08)_b_	5.22 (.05)_c_	6.44 (.04)_d_
Action intentions	1.90 (.13)_a_	2.93 (.09)_b_	3.59 (.06)_c_	4.75 (.07)_d_
Past action	1.00 (.13)_a_	1.24 (.08)_a_	2.44 (.09)_b_	4.64 (.15)_c_

The UK	Disengaged	Ambivalent	Partially internalised	Purely autonomous
Identification	4.42 (.29)_a_	5.27 (.07)_b_	6.05 (.05)_c_	5.79 (.11)_d_
Action intentions	1.77 (.14)_a_	3.19 (.07)_b_	4.35 (.07)_c_	3.90 (.15)_d_
Past action	1.03 (.20)_a_	2.09 (.11)_b_	3.96 (.13)_c_	3.25 (.26)_d_

*Note*: Subscripts denote where profiles are significantly different at *p* < .05.

#### Romania

**FIGURE 3:**
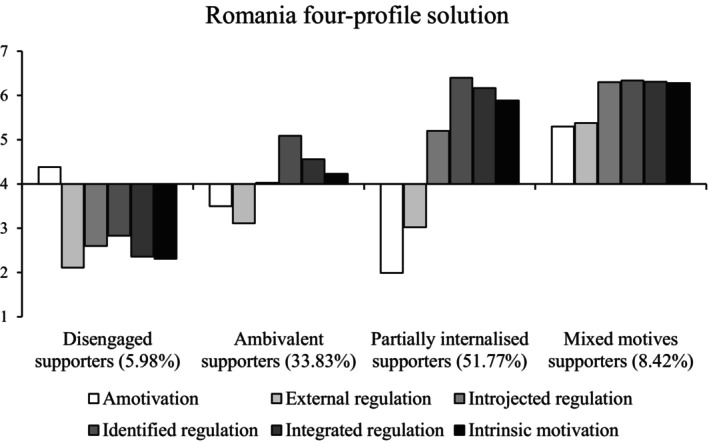
Profile means for each type of motivation in the four‐profile solution in Romania.

We concluded that the four‐profile solution had the best fit with the data (Figure 3); although the five‐profile solution had significant VLMR and lower AIC, BIC and adjusted BIC (Table 4), the fifth profile was theoretically very similar to another profile. This conclusion was supported by a higher entropy value for the four‐profile solution.

The four profiles were theoretically similar to four (of five) profiles identified in Study 1. First, *disengaged supporters* were low on all types of motivation and near the scale midpoint for amotivation. *Ambivalent supporters* scored near the midpoint for all motives, though they were somewhat more strongly driven by autonomous than controlled motivations. They were also near the midpoint for amotivation. Next, *partially internalised supporters* had high levels of autonomous motivation, moderate‐high levels of introjected regulation, low levels of external regulation and very low levels of amotivation compared to the other profiles. Finally, *mixed motives supporters* had very high levels of autonomous motivation and introjected regulation and moderate‐high levels of external regulation and amotivation. The purely autonomous profile (identified in Study 1) was not present. Disengaged supporters scored lowest on identification and action, followed by ambivalent supporters, then partially internalised supporters, with mixed motives supporters again scoring highest on all outcomes (Table 5).

#### Hungary

**FIGURE 4:**
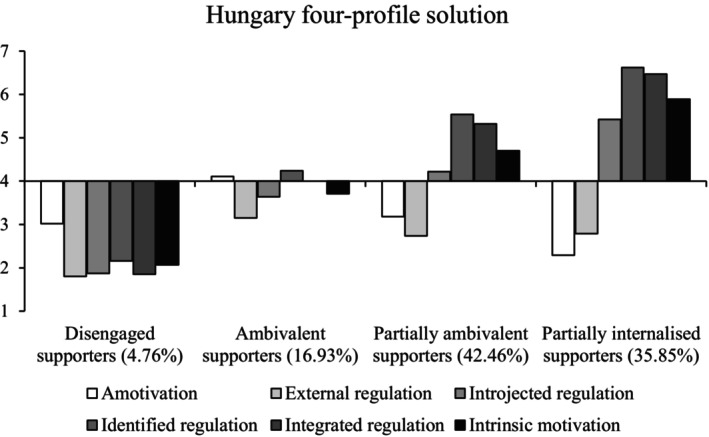
Profile means for each type of motivation in the four‐profile solution in Hungary.

We again concluded that the four‐profile solution had the best fit (Figure 4). The VLMR was only marginally significant for the five‐profile solution and the six‐profile solution, despite having a significant VLMR, contained multiple theoretically similar profiles. The larger entropy value also supported the four‐profile over the five‐profile solution (Table 4).

Three of the four profiles were similar to those found previously; *disengaged supporters* (low on all indicators), *ambivalent supporters* (near the scale midpoint on all indicators) and *partially internalised supporters* (high autonomous motivation, moderate‐high introjected regulation and low external regulation and amotivation). The new profile was characterised by moderate‐to‐high levels of autonomous motivations, midpoint levels of introjected regulation and somewhat low external regulation and amotivation. This profile presented a ‘middle ground’ between ambivalent and partially internalised supporters and was thus labelled as *partially ambivalent*. Mixed motives (Study 1 and Romania) and purely autonomous supporters (Study 1) were not present. Disengaged supporters scored lowest on all outcomes, followed by ambivalent, partially ambivalent and partially internalised supporters.

#### The UK

**FIGURE 5:**
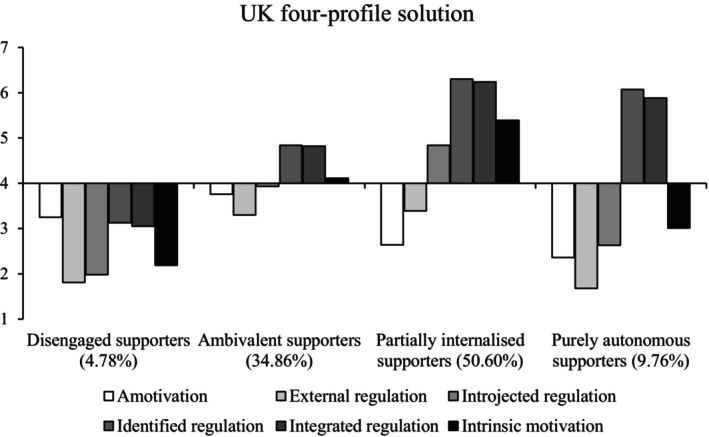
Profile means for each type of motivation in the four‐profile solution in the UK.

We again chose the four‐profile solution (Figure 5), as this solution had lower AIC, BIC and aBIC than the previous solutions and a significant VLMR (Table 4).

The same three profiles that were consistent across all studies were present in the UK sample; *disengaged supporters* (low on all indicators), *ambivalent supporters* (near the scale midpoint on all indicators, though autonomous motives were somewhat higher than controlled motives) and *partially internalised supporters* (high in autonomous motivations, moderate‐high introjected regulation, moderate‐low external regulation and low in amotivation). Finally, *purely autonomous supporters* (Study 1) were high in integrated and identified regulation and low in intrinsic motivation, introjected and external regulation and amotivation. The mixed motives profile (Study 1 and Romania) was not present. Disengaged supporters scored lowest on all outcomes, followed by ambivalent supporters, purely autonomous supporters and partially internalised supporters.

#### Multi‐group analysis

We conducted a multi‐group LPA following steps in Morin et al. (2016). We first established configural similarity, i.e., that the optimal number of profiles was the same in all countries. We thus conducted a multi‐group model with all three samples included, using country as the grouping variable. We then tested for structural similarity (mean indicator levels in each profile are equivalent between countries), dispersion similarity (within‐profile variance is equivalent between countries) and distributional similarity (the size of the profiles is equivalent between countries), increasingly constraining parameters of the model and comparing the AIC, BIC and aBIC values to determine whether fit improved or worsened between iterations (Table 6). All fit indices increased with every iteration, thus we retained the original configural similarity model and concluded that means, variances and class probabilities varied across countries. As the structure of the profiles was not equivalent, we did not test for explanatory similarity. The findings of the multi‐group analysis therefore suggest that the profiles of refugee supporters were different across our three samples. Although the analysis cannot tell us if any *specific* profiles were structurally similar across all (or some) countries, we conclude that disengaged, ambivalent and partially internalised supporters were present in all samples, but mixed motives, partially ambivalent and purely autonomous supporters were unique to Romania, Hungary and the UK, respectively.

**TABLE 6:** Fit statistics for cross‐national invariance tests.

	AIC	aBIC	BIC	Entropy
Configural	39,723.614	39,968.120	40,289.002	0.871
Structural	40,138.610	40,266.915	40,435.299	0.824
Dispersion	40,247.097	40,346.351	40,476.610	0.822
Distributional	40,291.970	40,376.699	40,487.896	0.823

### Discussion

We consistently found that members of profiles characterised by autonomous motivation alone were more strongly identified and committed to action than those low or ambivalent on all motives, as predicted (H1). Consistent with our pre‐registration for Study 2 but contrary to our initial hypotheses, profiles characterised by *both* autonomous and controlled motivation were again highest on identification and action (H2). There were no profiles where controlled motivation was high, but autonomous motivation was low (per H3).

The prediction that low scores on all motives would be associated with high levels of amotivation was not supported, as no clear patterns emerged (H4). Although ambivalent and disengaged profiles tended to score higher on amotivation than more highly engaged profiles (e.g., partially internalised, purely autonomous), the disengaged profiles in Hungary and the UK were still relatively low on amotivation (lower than the scale midpoint). Unexpectedly, the mixed motives profile in Romania was the only profile that scored highly on amotivation (H5). It may be that responses to one of the items (‘I wonder why I engage in this behaviour; in fact, I don't think that it changes anything’) reflected feelings of despair or low efficacy to effect change (see Table S9) rather than the absence of motivation or investment theorised by Ryan and Deci (2000).

Finally, H6 was generally supported, as supporters higher in autonomous motivation tended to also indicate that they experienced collective action as intrinsically more enjoyable than those lower in autonomous motivation. However, this was not the case for the purely autonomous group in the UK, who scored highly on integrated and identified regulation but were low in intrinsic motivation. This suggests that this particular group was driven only by concern for the cause and a desire to improve outcomes for refugees, but did not draw any satisfaction or enjoyment from participating in collective action. Although intrinsic motivation is considered the most authentic form of motivation for individual goals (Ryan & Deci, 2000), this may not be the case for collective goals where intrinsic motivation reflects a motive of personal enjoyment and satisfaction. Indeed, the satisfaction obtained from acting for a good cause, which could be considered as a form of intrinsic enjoyment (Van der Linden, 2018) may be more closely aligned with the introjected desire to feel good about oneself than with the goal‐focused motives of identified and integrated regulation. Furthermore, identity and goals‐based motives (identified and integrated regulation) were more highly endorsed as a motivator for action compared to intrinsic motivation, suggesting that intrinsic enjoyment is less important as a motivator of collective action compared to internalised goals (Koestner et al., 1996).

Cross‐national analysis revealed similarities and differences in the profiles across national contexts. Disengaged, ambivalent and partially internalised profiles were present in all samples and made up a similar proportion of each (except for Hungary, where some of these were partially ambivalent). However, Romania had a small group of mixed motives supporters, while the UK had a small group of purely autonomous supporters; Hungary had neither. There are key socio‐political differences between these countries which may explain differences in the profile structure. Romania has a history of accepting refugees dating back to World War II and its government has been less hostile towards refugees relative to other European countries (Balsam, 2023). Furthermore, Romania and Ukraine are geographically close and share a Christian Orthodox religion (Stan et al., 2023), which may lead Romanians to experience a greater sense of solidarity with Ukrainian refugees and ultimately result in some supporters who have both an internalised desire to support refugees but are also impacted by external norms and expectations of providing support. Thus, we found some supporters with mixed motives in our Romanian sample. In contrast, foreign policy in the UK has historically been unwelcoming towards refugees and negative attitudes towards refugees are high (Holloway et al., 2019), which may result in lower external pressure to support refugees. In the UK sample, we did not find any supporters who were high on external regulation, but we found some supporters who acted for purely internalised reasons, as those who take action may do so with minimal expectations of external reward. Meanwhile, greater ambivalence in motives among the Hungarian sample may similarly be a result of prior hostile attitudes towards refugees and anti‐immigrant campaigns by the government (Barna & Koltai, 2019), which may explain the low levels of external regulation throughout all subgroups in this sample. Nevertheless, public opinion towards refugees in Hungary was much more favourable towards Ukrainians than Middle Eastern refugees in the prior decade (Pepinsky et al., 2022) and indeed a large proportion of our sample demonstrated a high degree of internalised motivation to support refugees which may reflect feelings of solidarity with culturally similar (e.g., predominantly white Christian) Ukrainians. Although we can only speculate on causal factors, the differences between supporters in each country highlight the importance of considering the socio‐political context in which motivation develops. However, there are some limitations to these comparisons, as there are key demographic differences between samples (e.g., proportion of students).

## GENERAL DISCUSSION

Why do people take solidarity‐based action to support refugees? Perspectives on performative allyship and slacktivism suggest that some supporters take low‐effort actions for self‐serving reasons, while others take more meaningful action. However, this debate has not yet examined the underlying quality of motivation using statistical approaches suitable for detecting mixed motives. We found evidence to suggest that supporters of refugees differ based on the combinations of motives underlying their support and across contexts in which they live (see Table 7 for an overview). Some supporters were disengaged or ambivalent, some had purely autonomous motives, but some also had mixed motives, contrary to the continuum structure of self‐determined motivation (Ryan & Deci, 2000). In all four samples, the majority of supporters were driven by both autonomous motives and more self‐serving motives like those outlined in the slacktivism perspective (Skoric, 2012) to alleviate guilt or feel good about oneself. Unexpectedly, people with mixed motives consistently reported stronger identification with refugee supporters and higher engagement in action than those with autonomous motives alone (contrary to Geiser et al., 2014; Levesque‐Côté et al., 2021). Thus, a combination of motives is associated with the most positive outcomes and controlled motives appear conducive to action as long as autonomous motives are also present.

**TABLE 7:** Summary of all profiles in Study 1 and 2.

	Disengaged	Ambivalent	Partially ambivalent	Purely autonomous	Partially internalised	Mixed motives
Study 1	✔	✔		✔	✔	✔
Study 2 Romania	✔	✔			✔	✔
Study 2 Hungary	✔	✔	✔		✔	
Study 2 UK	✔	✔		✔	✔	
Autonomous motivation	Low	Neutral	Neutral‐high	High	High	High
Introjected regulation	Low	Neutral	Neutral‐high	Low	High	High
External regulation	Low	Neutral	Low	Low	Low	High
Outcomes	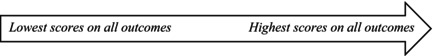					

Contrary to slacktivism and virtue‐signalling accounts of action, we did not find any profiles characterised by controlled motivation alone – and only small groups in some samples were high in external regulation (mixed motives supporters). Thus, we consistently did not find any supporters whose action was solely driven by impression management or similar self‐serving motives; although these motives are present among refugee supporters, we found no evidence that there are supporters who act purely, or even mainly, for performative reasons. This provides further nuance to discussions of performative allyship (Kutlaca & Radke, 2023; Radke et al., 2020) and suggests that the self‐focused motives identified in this literature co‐occur with motives focusing on the plight of the disadvantaged group. However, it is possible that people driven purely by external motives would not self‐identify as a supporter and thus were not captured in our sample, or that participants under‐reported external regulation, as those who are susceptible to impression management motives may also engage in socially desirable responding. Thus, future research is needed to address this limitation.

We found that controlled motivation was positively associated with identification and collective action, as supporters high in controlled motivation (when paired with high autonomous motivation) tended to report greater identification and commitment to action. Using a person‐centred approach allows us to examine how motives co‐occur within people, showing that controlled motivation can be a positive force for collective action when autonomous motivation is also present. In contrast, previous findings have shown that having both types of motivation can lead to moderate levels of action (Geiser et al., 2014; Levesque‐Côté et al., 2021), suggesting that the presence of controlled motivation diminishes the positive effects of autonomous motivation. One intriguing possibility is that controlled motivation may play a different role when pursuing group‐based rather than individual goals. In this context, the external influences whose norms and expectations control one's behaviour are other ingroup members whose approval we seek *because* we identify with them (Hogg & Reid, 2006) and thus are not truly external to one's (social) identity. Thus, when autonomous motivation (i.e., a genuine commitment to the cause) is also present, controlled motivation may be a positive force for action because it is grounded in one's commitment to the group.

However, research using variable‐centred approaches has shown that controlled motivation predicts decreases in identification and collective action over time (Yip et al., 2024). Furthermore, external regulation has been found to predict burnout in environmental activists (Sheldon et al., 2016), while introjected regulation can have positive effects on behaviour, but negative effects on wellbeing (Ng et al., 2012) and promote behaviour in the short‐term, but not in the long‐term (Pelletier et al., 2001). It may be that controlled motivation promotes short‐term involvement in action but can be detrimental over time by shifting the focus to external factors (e.g., ingroup approval) and away from the group's goals, or may lead to other detrimental effects (e.g., on wellbeing). Further research is needed to clarify the role of controlled motivation and understand its long‐term impacts on collective action and to establish causal relationships.

Partially internalised supporters were consistently the largest profile, comprising nearly half of all samples. Thus, the majority of supporters were high in both introjected regulation (e.g., guilt/pride) and autonomous motives (internalised goals), replicating the association between these motives found in previous research (Howard et al., 2017; Yip et al., 2024). This association may be because those who care about the cause and hold it as personally important are more likely to feel guilt or shame (Table S9) if they do not act, as they are failing to meet their own standards. Furthermore, highly autonomous supporters identify strongly with the opinion‐based group, meaning that their judgement of themselves (e.g., ‘I am a good person’) is likely to hinge on adherence to the group's norms. Thus, it may be that the continuum structure outlined in self‐determination theory is less applicable in the context of collective action and the role of introjected regulation in this context may be different to its role in individual self‐regulation (as discussed above). It is also notable that introjected regulation was strongly correlated with both autonomous motivation and external regulation (Table S9), suggesting that introjected regulation should be conceptualised as a middle ground between autonomous and controlled motives (Howard et al., 2017, 2020) rather than a type of controlled motivation.

## CONCLUSION

What drives advantaged group members to take action to support the millions of refugees fleeing conflict and persecution around the world? Understanding the motives behind these actions and how they vary across socio‐political contexts is key to understanding how to promote and sustain support for refugees. We found that there are qualitatively different types of supporters distinguishable by the combinations of motives underlying their support. We found no evidence that any supporters act purely for self‐serving reasons. Instead, most supporters are driven by a combination of their internalised goals aligning with the cause and introjected factors such as guilt. Supporters with mixed motives are more committed to action than any other group, suggesting that both quality (i.e., the presence of autonomous motivation) and quantity (multiple or mixed motives) are important for action.

## AUTHOR CONTRIBUTIONS

**Lisette Yip:** Conceptualization; investigation; writing – original draft; methodology; writing – review and editing; project administration; formal analysis. **Emma F. Thomas:** Conceptualization; investigation; methodology; writing – review and editing; funding acquisition. **Ana‐Maria Bliuc:** Investigation; writing – review and editing. **Mihaela Boza:** Investigation; writing – review and editing. **Anna Kende:** Writing – review and editing; investigation. **Morgana Lizzio‐Wilson:** Writing – review and editing. **Gerhard Reese:** Writing – review and editing; investigation. **Laura G. E. Smith:** Writing – review and editing; investigation.

## CONFLICT OF INTEREST STATEMENT

The author(s) declared that there were no conflicts of interest with respect to the research, authorship, or publication of this article.

## Supporting information


Appendix S1.

## Data Availability

The data that support the findings of this study are openly available in OSF at https://osf.io/un4a5/?view_only=c0e007d9531043a9817808d0ae9a7fc9.
