# Supplementary material for: Motivations to engage in collective action: A latent profile analysis of refugee supporters
Source: Br J Soc Psychol. 2024 Jul 9;64(1):e12786. doi: 10.1111/bjso.12786 (PMC11600512; doi:10.1111/bjso.12786)
Supplement: Supplementary file 1 — Appendix S1. [file BJSO-64-0-s001.docx]

**Supplementary Materials**

**Predictors of Profile Membership**

If there are qualitative differences in the underlying pattern of motivation in support for refugees, it stands to reason that such motivations would also be shaped by the emotional responses that people have to the disadvantage experienced by refugees. Emotional reactions to injustice have been shown to be precursors to collective action (Thomas et al., 2009; van Zomeren et al., 2008). We first examine whether different emotions are associated with different motives for engaging in collective action. Sympathy is an other-focussed emotion as the emphasis is on the experience of the disadvantaged outgroup (Harth et al., 2008). Similarly, anger is other-focussed as it targets an offending outgroup or perpetrator who is blamed for causing harm (though in some cases anger can be self-focussed if it is targeted at the ingroup; Leach et al., 2006). In contrast, guilt is self-focussed as the emphasis is on the relative advantage of the ingroup, and how the actions of the ingroup have inflicted harm (Harth et al., 2008). Other-focussed emotions of sympathy and anger are characterised by a concern for the wellbeing of the disadvantaged group (Thomas et al., 2009) and thus may be associated with autonomous motivation. In contrast, guilt centres one’s own experience rather than that of the disadvantaged group, and may lead to a motive to alleviate one’s own discomfort, and thus may be associated with controlled motivation. Sympathy (Harth et al., 2008) and anger (Leach et al., 2006) have been found to be greater predictors of action in support of disadvantaged groups when compared with guilt.

**Study 1**

We expected that profiles higher in autonomous motivation but lower in controlled motivation would be predicted by higher levels of other-focused emotions including sympathy an outrage. We expected that profiles higher in controlled motivation but lower in autonomous motivation would be predicted by higher levels of guilt. We did not have specific predictions about the role of hope, but included it as a predictor in the analysis as it was included in our measures and has been implicated as a predictor of collective action engagement (e.g., Greenaway et al., 2016; Wlodarczyk et al., 2017).

*Measures*

*Emotions.* Participants were asked to what extend they felt sympathetic, compassionate, guilty, responsible, angry, outraged, hope, and optimism when considering the plight of Syrian refugees. Each pair of items was averaged to create composite scores of sympathy, guilt, outrage, and hope.

**Results**

We used the AUXILIARY option in Mplus to examine the predictors of profile membership. We entered four emotions (guilt, outrage, sympathy, and hope) as predictors of the profiles. Table 1 displays how each emotion predicts likelihood of an individual belonging to each of the five profiles, compared to other profiles.

Disengaged supporters were lower in sympathy and hope relative to most other profiles (see Table 1), consistent with predictions for sympathy, and suggesting that lower hope is associated with a lack of motivation. Ambivalent supporters were lower in sympathy relative to purely autonomous, partially internalised, and mixed motives supporters, and higher in hope relative to disengaged supporters. Purely autonomous supporters were higher in sympathy than disengaged or ambivalent supporters, higher in outrage than disengaged supporters, and lower in guilt than partially internalised and mixed motives supporters. Partially internalised supporters were higher in sympathy and guilt than ambivalent or purely autonomous supporters; thus, the hypothesis that higher controlled motivation will be associated with guilt was supported, but higher controlled motivation was not associated with lower sympathy. Further, contrary to predictions, guilt was not associated with lower autonomous motivation. Mixed motives supporters were higher in hope than partially internalised supporters and reported greater guilt and hope relative to purely autonomous supporters. They were also higher in outrage compared to all except the purely autonomous group. Thus, the hypothesis that profiles higher in autonomous motivation but lower in controlled motivation would be predicted by higher levels of outrage was not supported.

**Table 1**

*Study 1 Profile Predictors: Likelihood of being in the first listed profile relative to the second*

|  | Ambivalent vs Disengaged | | Purely autonomous vs Disengaged | | Partially internalised vs Disengaged | | Mixed motives vs Disengaged | | Purely autonomous vs Ambivalent | |
| --- | --- | --- | --- | --- | --- | --- | --- | --- | --- | --- |
|  | Coeff. *(SE)* | Odds Ratio | Coeff. *(SE)* | Odds Ratio | Coeff. *(SE)* | Odds Ratio | Coeff. *(SE)* | Odds Ratio | Coeff. *(SE)* | Odds Ratio |
| Sympathy | -0.14 (.16) | 0.873 | 0.59 (.16) *** | 1.810 | 0.88 (.17)*** | 2.415 | 0.66 (.29)* | 1.929 | 0.73 (.16)*** | 2.073 |
| Guilt | 0.02 (.15) | 1.023 | 0.06 (.13) | 1.063 | 0.37 (.13)* | 1.447 | 0.35 (.16)* | 1.421 | 0.04 (.10) | 1.040 |
| Outrage | 0.02 (.15) | 1.125 | 0.13 (.09) | 1.142 | 0.21 (.09)* | 1.236 | 0.30 (.12)* | 1.355 | 0.02 (.08) | 1.015 |
| Hope | 0.29 (.14)* | 1.339 | 0.27 (.13)* | 1.310 | 0.34 (.13)** | 1.404 | 0.58 (.15)*** | 1.781 | -0.02 (.10) | 0.978 |
|  | Partially internalised vs Ambivalent | | Mixed motives vs Ambivalent | | Partially internalised vs Purely autonomous | | Mixed motives vs Purely autonomous | | Mixed motives vs Partially internalised | |
|  | Coeff. *(SE)* | Odds Ratio | Coeff. *(SE)* | Odds Ratio | Coeff. *(SE)* | Odds Ratio | Coeff. *(SE)* | Odds Ratio | Coeff. *(SE)* | Odds Ratio |
| Sympathy | 1.02 (.17)*** | 2.766 | 0.79 (.29)** | 2.209 | 0.29 (.14)* | 1.334 | 0.06 (.26) | 1.065 | -0.23 (.26) | 0.799 |
| Guilt | 0.35 (.10)*** | 1.415 | 0.33 (.13)* | 1.389 | 0.31 (.07)*** | 1.361 | 0.29 (.11)** | 1.336 | -0.02 (.10) | 0.982 |
| Outrage | 0.09 (.08) | 1.099 | 0.19 (.12) | 1.205 | 0.08 (.06) | 1.082 | 0.17 (.10) | 1.187 | 0.09 (.10) | 1.097 |
| Hope | 0.05 (.10) | 1.048 | 0.29 (.14)* | 1.330 | 0.07 (.07) | 1.071 | 0.31 (.11)** | 1.359 | 0.24 (.10)* | 1.269 |

*p* < .05; ***p* < .01; ****p* < .001

**Study 2**

We added measures of group efficacy and moral conviction, highlighted in Agostini and van Zomeren (2021)’s extension of the social identity model of collective action (SIMCA) as key predictors of collective action, in addition to emotional pathways (e.g., anger). We expected that higher moral conviction would predict membership of profiles higher in autonomous motivation, as moral convictions represent strongly held personal values and ideals and thus lead to autonomous, internalised motivation to act in accordance with one’s moral stance (Skitka, 2010). We expected that higher efficacy would also predict membership of profiles higher in autonomous motivation, as self-determination theory posits that people are more likely to participate in and internalise (i.e., experience as autonomous) activities in which they expect to be competent (Deci & Ryan, 1985).

*Measures*

*Emotions.* The same measures of sympathy, guilt, outrage, and hope were used, with an additional two-item measure of despair: ‘Considering the plight of Ukrainian refugees, I feel: Despair/Depressed’.

*Group efficacy.* Two items measured group efficacy and were averaged to create a single score: ‘Together we can improve the outcomes for Ukrainian refugees’ and ‘Together we can make a positive difference for Ukrainian refugees.’

*Moral conviction*. Three items assessed participants’ moral conviction regarding their stance on Ukrainian refugees, e.g., ‘My feelings about Ukrainian refugees are a reflection of my core moral beliefs and convictions.’ These three items were averaged to create a composite score.

**Results**

*Romania*

Table 2 displays profile predictors for the Romanian data. Disengaged supporters had lower levels of efficacy, guilt, and moral conviction than most other profiles. Ambivalent supporters were lower in guilt and moral conviction than partially internalised and mixed motives supporters, but higher in efficacy compared to the disengaged group. Partially internalised supporters were higher in efficacy than all other profiles, and higher in sympathy, guilt, and moral conviction relative to disengaged or ambivalent supporters. Membership of the mixed motives profile was predicted by higher guilt and moral conviction compared to all other profiles, higher hope than the disengaged group, and higher despair than the ambivalent group. Membership was also predicted by lower levels of efficacy than partially internalised supporters. Further, contrary to Study 1, outrage did not emerge as a significant predictor of any profile memberships.

*Hungary*

Table 3 displays profile predictors for Hungary. Membership of the disengaged profile was predicted by lower efficacy and moral conviction relative to the other profiles. Membership of the ambivalent group was predicted by lower efficacy and moral conviction than partially ambivalent and partially internalised profiles, but higher moral conviction than the disengaged group. Ambivalent supporters had higher levels of despair relative to the disengaged supporters. Partially ambivalent supporters had higher sympathy, efficacy and moral conviction than ambivalent supporters, but lower sympathy and moral conviction than partially internalised supporters. Membership of the partially internalised group was predicted by higher sympathy and moral conviction than all other profiles, and higher efficacy than all but partially ambivalent supporters. Similar to the Romanian sample, outrage did not emerge as a significant predictor of any profile memberships. However, in the Hungarian sample, guilt also did not predict any profiles.

*UK*

Table 4 displays profile predictors for the UK. Disengaged supporters were lower in outrage and hope than all other profiles. Membership of the ambivalent group was predicted by higher efficacy, outrage and hope than disengaged supporters, and lower sympathy than purely autonomous supporters. Membership of the partially internalised profile was predicted by higher levels of guilt than purely autonomous supporters. There were no significant predictors separating ambivalent and partially internalised supporters. In the UK sample, moral conviction and despair did not predict any profile memberships.

**Discussion**

We found somewhat inconsistent effects for how profile membership was predicted by emotions. Outrage, a well-established predictor of action (van Zomeren, 2021; van Zomeren et al., 2008), had no effect on profile membership in Romania or Hungary. In the UK, membership of the disengaged group was predicted by lower levels of outrage compared to all other groups, suggesting that the least outraged supporters were the least motivated to take action. There were more consistent effects for sympathy, which predicted membership of the partially internalised group over most other groups (except purely autonomous and mixed motives) in all three nations. Thus, we only partially replicated the finding that more highly autonomous profiles were characterised by other-focussed emotions, demonstrating greater concern for the plight of the disadvantaged group. We also found that higher levels of guilt predicted membership of profiles more strongly characterised by controlled motivation in Romania and the UK, but not in Hungary.

Efficacy also tended to predict membership of more highly autonomous (and thus more active) groups over less autonomous groups, but some effects were non-significant. However, the mixed motives group in Romania, despite being the most committed to taking action, was predicted by lower efficacy than the partially internalised group and was no different to the ambivalent and disengaged groups. It may be that the presence of external regulation as a motivator acted as a buffer against the demotivating effects of lower group efficacy, as external goals – such as impression management – provide an incentive to continue acting even when there is low efficacy to achieve the group goal (i.e., improving the plight of Ukrainian refugees). Moral conviction consistently predicted membership of more highly autonomous groups in Hungary and Romania – indeed, those who value the cause and feel that it is personally important should hold stronger moral convictions – but it had no effects in the UK.

**Table 2**

*Study 2 (Romania) Profile Predictors: Likelihood of being in the first listed profile relative to the second*

|  | Ambivalent vs Disengaged | | Partially internalised vs Disengaged | | Mixed motives vs Disengaged | | Partially internalised vs Ambivalent | | Mixed motives vs Ambivalent | | Mixed motives vs Partially internalised | |
| --- | --- | --- | --- | --- | --- | --- | --- | --- | --- | --- | --- | --- |
|  | Coeff. *(SE)* | Odds Ratio | Coeff. *(SE)* | Odds Ratio | Coeff. *(SE)* | Odds Ratio | Coeff. *(SE)* | Odds Ratio | Coeff. *(SE)* | Odds Ratio | Coeff. *(SE)* | Odds Ratio |
| Sympathy | 0.18 (.19) | 1.198 | 0.61 (.24)** | 1.847 | 0.30 (.35) | 1.355 | 0.43 (.16)** | 1.542 | 0.12 (.30) | 1.132 | -0.31 (.29) | 0.734 |
| Guilt | 0.47 (.25) | 1.598 | 0.78 (.27)** | 2.189 | 1.57 (.30)*** | 4.821 | 0.32 (.11)** | 1.370 | 1.10 (.18)*** | 3.017 | 0.79 (.15)*** | 2.203 |
| Outrage | 0.05 (.13) | 1.053 | -0.01 (.14) | 0.991 | -0.08 (.17) | 0.928 | -0.06 (.07) | 0.941 | -0.13 (.12) | 0.881 | -0.07 (.11) | 0.936 |
| Hope | 0.28 (.17) | 1.324 | 0.36 (.18) | 1.426 | 0.81 (.37)* | 2.239 | 0.07 (.09) | 1.077 | 0.53 (.33) | 1.692 | 0.45 (.32) | 1.571 |
| Despair | 0.09 (.20) | 1.090 | 0.26 (.21) | 1.292 | 0.50 (.27) | 1.648 | 0.17 (.09) | 1.186 | 0.41 (.19)* | 1.512 | 0.24 (.17) | 1.275 |
| Efficacy | 0.46 (.21)* | 1.580 | 1.30 (.25)*** | 3.661 | 0.62 (.36) | 1.867 | 0.84 (.16)*** | 2.318 | 0.17 (.30) | 1.182 | -0.67 (.29)* | 0.510 |
| Moral conviction | 0.18 (.19) | 1.191 | 0.89 (.23)*** | 2.430 | 1.62 (.35)*** | 5.054 | 0.71 (.16)*** | 2.040 | 1.45 (.31)*** | 4.242 | 0.73 (.28)** | 2.079 |

*p* < .05; ***p* < .01; ****p* < .001

**Table 3**

*Study 2 (Hungary) Profile Predictors: Likelihood of being in the first listed profile relative to the second*

|  | Ambivalent vs Disengaged | | Partially ambivalent vs Disengaged | | Partially internalised vs Disengaged | | Partially ambivalent vs Ambivalent | | Partially internalised vs Ambivalent | | Partially internalised vs Partially ambivalent | |
| --- | --- | --- | --- | --- | --- | --- | --- | --- | --- | --- | --- | --- |
|  | Coeff. *(SE)* | Odds Ratio | Coeff. *(SE)* | Odds Ratio | Coeff. *(SE)* | Odds Ratio | Coeff. *(SE)* | Odds Ratio | Coeff. *(SE)* | Odds Ratio | Coeff. *(SE)* | Odds Ratio |
| Sympathy | -0.33 (.25) | 0.718 | 0.16 (.26) | 1.169 | 1.06 (.38)** | 2.887 | 0.49 (.15)** | 1.629 | 1.39 (.31)*** | 4.022 | 0.90 (.29)** | 2.469 |
| Guilt | -0.002 (.21) | 0.998 | 0.05 (.21) | 1.048 | 0.22 (.25) | 1.250 | 0.05 (.12) | 1.051 | 0.23 (.17) | 1.253 | 0.18 (.13) | 1.192 |
| Outrage | 0.22 (.19) | 1.248 | 0.22 (.20) | 1.241 | 0.27 (.23) | 1.315 | -0.006 (.11) | 0.994 | 0.05 (.16) | 1.054 | 0.06 (.12) | 1.059 |
| Hope | 0.29 (.21) | 1.337 | 0.34 (.21) | 1.399 | 0.51 (.23)* | 1.663 | 0.05 (.10) | 1.046 | 0.22 (.13) | 1.244 | 0.17 (.11) | 1.189 |
| Despair | 0.46 (.22)* | 1.586 | 0.40 (.23) | 1.485 | 0.26 (.25) | 1.301 | -0.07 (.15) | 0.936 | -0.20 (.18) | 0.820 | -0.13 (.12) | 0.876 |
| Efficacy | 0.28 (.25) | 1.324 | 0.72 (.28)* | 2.045 | 0.96 (.35)** | 2.598 | 0.44 (.15)** | 1.545 | 0.67 (.25)** | 1.963 | 0.24 (.27) | 1.271 |
| Moral conviction | 0.55 (.21)** | 1.738 | 1.02 (.24)*** | 2.760 | 2.39 (.35)*** | 10.869 | 0.46 (.16)** | 1.588 | 1.83 (.30)*** | 6.252 | 1.37 (.27)*** | 3.937 |

*p* < .05; ***p* < .01; ****p* < .001

**Table 4**

*Study 2 (UK) Profile Predictors: Likelihood of being in the first listed profile relative to the second*

|  | Ambivalent vs Disengaged | | Partially internalised vs Disengaged | | Purely autonomous vs Disengaged | | Purely autonomous vs Ambivalent | | Partially internalised vs Ambivalent | | Purely autonomous vs Partially internalised | |
| --- | --- | --- | --- | --- | --- | --- | --- | --- | --- | --- | --- | --- |
|  | Coeff. *(SE)* | Odds Ratio | Coeff. *(SE)* | Odds Ratio | Coeff. *(SE)* | Odds Ratio | Coeff. *(SE)* | Odds Ratio | Coeff. *(SE)* | Odds Ratio | Coeff. *(SE)* | Odds Ratio |
| Sympathy | 0.27 (.46) | 1.306 | 1.56 (.75)* | 4.771 | 1.45 (.65)* | 4.248 | 1.18 (.48)* | 3.252 | 1.30 (.67) | 3.652 | -0.12 (.50) | 0.890 |
| Guilt | 0.45 (.34) | 1.570 | 0.55 (.36) | 1.729 | 0.19 (.38) | 1.206 | -0.26 (.18) | 0.768 | 0.10 (.15) | 1.101 | -0.36 (.17)* | 0.697 |
| Outrage | 0.51 (.24)* | 1.668 | 0.75 (.27)** | 2.113 | 0.77 (.31)* | 2.161 | 0.26 (.21) | 1.296 | 0.24 (.14) | 1.267 | 0.02 (.18) | 1.023 |
| Hope | 1.23 (.43)** | 3.404 | 1.64 (.48)** | 5.168 | 1.34 (.49)** | 3.829 | 0.12 (.26) | 1.125 | 0.42 (.25) | 1.518 | -0.30 (.20) | 0.741 |
| Despair | 0.24 (.30) | 1.269 | 0.16 (.35) | 1.169 | 0.07 (.36) | 1.069 | -0.17 (.25) | 0.842 | -0.08 (.23) | 0.922 | -0.09 (.18) | 0.914 |
| Efficacy | 0.92 (.44)* | 2.511 | 1.43 (.80) | 4.185 | 1.82 (.65)** | 6.181 | 0.90 (.53) | 2.462 | 0.51 (.72) | 1.667 | 0.39 (.49) | 1.477 |
| Moral conviction | 0.09 (.30) | 1.098 | 0.47 (.77) | 1.592 | 0.03 (.54) | 1.028 | -0.07 (.50) | 0.937 | 0.37 (.76) | 1.450 | -0.44 (.40) | 0.646 |

*p* < .05; ***p* < .01; ****p* < .001

**Specific Actions as Outcomes of Profile Membership**

As a supplementary analysis, we examined whether there were any differences in how the profiles predicted intentions and self-reported behaviour for specific collective actions (Table 5). These results mostly followed the overall pattern of the composite measures of intentions and behaviours, except that partially internalised supporters reported significantly higher intentions to volunteer than mixed motives supporters, and there was no difference between mixed motives supporters and purely autonomous supporters on intentions to volunteer or post on social media. For self-reported behaviour, only partially internalised supporters donated significantly more than other groups. Partially internalised supporters also volunteered more than purely autonomous and ambiguous supporters, but no other groups were significantly different from each other.

**Table 5**

*Study 1: Individual Actions (Intentions and Past Action) as Outcomes of Profile Membership*

| Intentions *M (SE)*  Past action % | Disengaged | Ambivalent | Purely internalised | Partially internalised | Mixed motives |
| --- | --- | --- | --- | --- | --- |
| Sign petition | 3.92 (.21)_a_  14.2%_a_ | 4.32 (.17)_a_  10.9%_a_ | 5.28 (.09)_b_  28.5%_b_ | 5.90 (.06)_c_  42.0%_c_ | 5.81 (.16)_c_  49.5%_c_ |
| Donate | 3.55 (.21)_a_  7.8%_abc_ | 3.98 (.17)_ab_  9.4%_adef_ | 4.33 (.10)_b_  16.6%_bcdf_ | 5.09 (.08)_c_  25.2%_g_ | 5.02 (.19)_c_  22.5%_cefg_ |
| Post on social media | 2.91 (.23)_a_  19.2%_ab_ | 3.06 (.19)_a_  7.7%_a_ | 4.19 (.12)_b_  36.6%_c_ | 4.71 (.10)_c_  46.5%_d_ | 4.68 (.24)_bc_  40.7%_bcd_ |
| Volunteer | 4.12 (.22)_ab_  15.3%_abcd_ | 3.87 (.17)_a_  10.1%_aef_ | 4.82 (.09)_c_  16.1%_beg_ | 5.16 (.08)_d_  24.3%_ch_ | 4.65 (.19)_bc_  13.4%_dfgh_ |

Subscripts denote where profiles are significantly different at *p* < .05.

We repeated the same analysis for Study 2. In Romania (Table 6), the same ranking we observed for composite scores (disengaged supporters scoring lowest, and mixed motives scoring highest) was observed for almost all individual actions, though for some cases adjacently ranked profiles were not significantly different from each other. However, partially internalised supporters intended to talk to friends and family more than mixed motives supporters, and donated more. In Hungary (Table 7), the ranking of profiles was also consistent between composite scores and all individual actions (with some non-significant differences), except for self-reported volunteering, where disengaged supporters volunteered more than ambivalent supporters. In the UK (Table 8), many of the differences between profiles for specific actions were not significant, but there were no clear and significant deviations from the overall pattern.

**Table 6**

*Study 2 (Romania): Individual Actions (Intentions and Past Action) as Outcomes of Profile Membership*

| Intentions *M (SE)*  Past action % | Disengaged | Ambivalent | Partially internalised | Mixed motives |
| --- | --- | --- | --- | --- |
| Post on social media | 1.80 (.16)_a_  0.0%_a_ | 3.20 (.11)_b_  19.1%_b_ | 4.35 (.09)_c_  53.9%_c_ | 5.48 (.18)_d_  54.0%_c_ |
| Carry/display item | 1.67 (.14)_a_  2.2%_a_ | 2.91 (.10)_b_  9.6%_b_ | 3.98 (.09)_c_  29.9%_c_ | 5.48 (.17)_d_  48.8%_d_ |
| Social media profile | 1.77 (.15)_a_  0.0%_a_ | 2.88 (.10)_b_  13.5%_b_ | 3.84 (.09)_c_  40.6%_c_ | 5.51 (.18)_d_  51.4%_c_ |
| Sign petition | 2.48 (.23)_a_  36.8%_a_ | 4.36 (.11)_b_  78.4%_b_ | 5.89 (.06)_c_  97.7%_c_ | 5.66 (.16)_c_  90.0%_c_ |
| Talk to friends/family | 2.86 (.24)_a_  2.2%_a_ | 4.69 (.10)_b_  21.7%_b_ | 6.17 (.05)_c_  54.0%_c_ | 5.90 (.13)_d_  57.1%_c_ |
| Email to politician | 1.61 (.14)_a_  0.0%_a_ | 2.58 (.10)_b_  2.3%_b_ | 3.20 (.09)_c_  2.3%_b_ | 5.15 (.18)_d_  16.0%_c_ |
| Donate | 2.10 (.18)_a_  7.8%_a_ | 4.23 (.10)_b_  30.0%_b_ | 5.76 (.06)_c_  66.5%_c_ | 5.83 (.14)_c_  47.8%_d_ |
| Attend rally | 1.86 (.17)_a_  0.0%_a_ | 3.30 (.11)_b_  3.4%_b_ | 4.78 (.08)_c_  13.7%_c_ | 5.73 (.16)_d_ 40.6%_d_ |
| Volunteer | 2.10 (.19)_a_  4.5%_a_ | 3.95 (.11)_b_  13.9%_b_ | 5.77 (.06)_c_  49.9%_c_ | 6.17 (.12)_d_  54.1%_c_ |
| Offer a place to stay | 1.85 (.17)_a_  0.0%_a_ | 3.18 (.11)_b_  5.8%_b_ | 4.31 (.09)_c_  13.9%_c_ | 5.79 (.17)_d_  19.6%_c_ |

Subscripts denote where profiles are significantly different at *p* < .05.

**Table 7**

*Study 2 (Hungary): Individual Actions (Intentions and Past Action) as Outcomes of Profile Membership*

| Intentions *M (SE)*  Past action % | Disengaged | Ambivalent | Ambivalent-Partially internalised | Partially internalised |
| --- | --- | --- | --- | --- |
| Post on social media | 1.57 (.15)_a_  0.0%_a_ | 2.48 (.14)_b_  5.1%_b_ | 3.03 (.10)_c_  18.3%_b_ | 4.40 (.12)_d_  54.1%_c_ |
| Carry/display item | 1.60 (.15)_a_  2.9%_a_ | 2.50 (.13)_b_  5.0%_a_ | 2.99 (.09)_c_  14.5%_b_ | 4.07 (.11)_d_  53.5%_c_ |
| Social media profile | 1.72 (.17)_a_  5.1%_ab_ | 2.40 (.13)_b_  2.3%_a_ | 2.93 (.10)_c_  15.7%_b_ | 4.27 (.12)_d_  42.5%_c_ |
| Sign petition | 2.19 (.25)_a_  66.5%_a_ | 3.50 (.17)_b_  76.4%_a_ | 4.44 (.10)_c_  95.0%_b_ | 5.50 (.09)_d_  98.0%_b_ |
| Talk to friends/family | 3.73 (.30)_a_  6.7%_a_ | 4.56 (.13)_b_  11.9%_a_ | 5.46 (.07)_c_  22.0%_b_ | 6.64 (.04)_d_  42.5%_c_ |
| Email to politician | 1.42 (.12)_a_  0.0%_ab_ | 2.14 (.12)_b_  2.5%_acd_ | 2.42 (.09)_b_  0.6%_bc_ | 3.13 (.12)_c_  5.9%_d_ |
| Donate | 2.03 (.20)_a_  4.0%_a_ | 3.30 (.14)_b_  9.7%_a_ | 4.01 (.09)_c_  26.4%_b_ | 5.50 (.09)_d_  62.1%_c_ |
| Attend rally | 1.62 (.16)_a_  0.0%_a_ | 2.79 (.15)_b_  3.4%_b_ | 3.27 (.10)_c_  9.5%_c_ | 4.49 (.11)_d_  21.0%_d_ |
| Volunteer | 2.01 (.20)_a_  13.7%_a_ | 3.30 (.13)_b_  1.5%_b_ | 4.41 (.08)_c_  26.3%_a_ | 5.89 (.08)_d_  57.1%_c_ |
| Offer a place to stay | 1.43 (.12)_a_  2.7%_ab_ | 2.34 (.12)_b_  2.2%_ac_ | 2.56 (.09)_b_  4.4%_bc_ | 3.63 (.12)_c_  23.0%_d_ |

Subscripts denote where profiles are significantly different at *p* < .05.

**Table 8**

*Study 2 (UK): Individual Actions (Intentions and Past Action) as Outcomes of Profile Membership*

| Intentions *M (SE)*  Past action % | Disengaged | Ambivalent | Purely internalised | Partially internalised |
| --- | --- | --- | --- | --- |
| Post on social media | 1.50 (.17)_a_  9.6%_a_ | 2.68 (.12)_b_  11.5%_a_ | 3.43 (.26)_c_  39.4%_b_ | 4.07 (.12)_d_  50.2%_b_ |
| Carry/display item | 1.52 (.16)_a_  4.1%_a_ | 3.03 (.12)_b_  11.9%_ab_ | 3.17 (.23)_b_  23.5%_b_ | 4.32 (.11)_c_  44.8%_c_ |
| Social media profile | 1.74 (.20)_a_  0.0%_a_ | 2.93 (.12)_b_  15.4%_b_ | 3.33 (.24)_b_  22.9%_b_ | 4.29 (.11)_c_  43.7%_c_ |
| Sign petition | 2.33 (.32)_a_  48.5%_a_ | 4.14 (.14)_b_  79.9%_b_ | 5.53 (.18)_c_  93.3%_c_ | 5.74 (.07)_c_  95.5%_c_ |
| Talk to friends/family | 2.40 (.32)_a_  12.2%_a_ | 4.37 (.12)_b_  26.3%_a_ | 5.84 (.13)_c_  45.8%_b_ | 5.70 (.06)_c_  59.6%_b_ |
| Email to politician | 1.04 (.04)_a_  8.1%_ade_ | 2.65 (.11)_b_  4.0%_be_ | 3.13 (.23)_bc_  11.3%_abc_ | 3.57 (.11)_c_  10.8%_cd_ |
| Donate | 2.56 (.33)_a_  20.4%_a_ | 3.98 (.14)_b_  51.8%_b_ | 5.00 (.19)_c_  60.0%_bc_ | 5.37 (.08)_c_  72.4%_c_ |
| Attend rally | 1.34 (.13)_a_  0.0%_ab_ | 2.56 (.11)_b_  1.8%_cb_ | 3.02 (.22)_bc_  8.2%_acd_ | 3.44 (.11)_c_  5.8%_d_ |
| Volunteer | 1.62 (.18)_a_  6.2%_ade_ | 3.19 (.11)_b_  5.9%_be_ | 3.93 (.20)_c_  13.0%_abc_ | 4.40 (.09)_d_  13.7%_cd_ |
| Offer a place to stay | 1.33 (.12)_a_  0.0%_ab_ | 2.14 (.09)_b_  0.0%_cb_ | 2.40 (.19)_bc_  3.9%_acd_ | 2.70 (.09)_c_  2.8%_d_ |

Subscripts denote where profiles are significantly different at *p* < .05.

**Supplementary Tables**

**Table 9**

Correlations Between Motivation Types and Other Key Variables

|  |  | Intrinsic motivation | Integrated regulation | Identified regulation | Introjected regulation | External regulation | Amotivation | Sample mean |
| --- | --- | --- | --- | --- | --- | --- | --- | --- |
| Sympathy | Study 1 | - | .499*** | .403*** | .196*** | -.187*** | - | 6.20 (0.92) |
|  | Romania | .426*** | .411*** | .473*** | .359*** | .051 | -.180*** | 6.03 (0.94) |
|  | Hungary | .359*** | .507*** | .528*** | .341*** | -.066 | -.238*** | 5.86 (1.00) |
|  | UK | .299*** | .572*** | .563*** | .263*** | .002 | -.280*** | 6.19 (0.74) |
| Guilt | Study 1 | - | .274*** | .271*** | .265*** | -.057 | - | 3.91 (1.57) |
|  | Romania | .378*** | .389*** | .269*** | .410*** | .307*** | -.004 | 3.69 (1.36) |
|  | Hungary | .307*** | .343*** | .284*** | .363*** | .133*** | -.107** | 3.91 (1.30) |
|  | UK | .247*** | .280*** | .240*** | .307*** | .236*** | -.012 | 3.34 (1.36) |
| Outrage | Study 1 | - | .417*** | .296*** | .214*** | -.047 |  | 4.92 (1.81) |
|  | Romania | .138*** | .149*** | .089* | .080* | .018 | .022 | 4.056 (1.85) |
|  | Hungary | .216*** | .346*** | .331*** | .326*** | .012 | -.091* | 4.97 (1.51) |
|  | UK | .111* | .297*** | .351*** | .145** | .002 | -.060 | 5.11 (1.44) |
| Hope | Study 1 | - | .126*** | .133*** | .040 | .056 | - | 4.22 (1.50) |
|  | Romania | .319*** | .276*** | .299*** | .303*** | .151*** | -.043 | 5.39 (1.29) |
|  | Hungary | .305*** | .307*** | .239*** | .248*** | .121*** | -.038 | 4.36 (1.34) |
|  | UK | .276*** | .264*** | .267*** | .204*** | .108* | -.144** | 4.77 (1.15) |
| Despair | Study 1 | - | - | - | - | - | - | - |
|  | Romania | .328*** | .329*** | .311*** | .362*** | .218*** | -.003 | 4.30 (1.46) |
|  | Hungary | .203*** | .266*** | .237*** | .260*** | .124*** | -.030 | 4.79 (1.31) |
|  | UK | .148*** | .284*** | .285*** | .255*** | .097* | .026 | 4.17 (1.34) |
| Efficacy | Study 1 | - | - | - | - | - | - | - |
|  | Romania | .554*** | .544*** | .609*** | .365*** | .042 | -.303*** | 5.94 (1.08) |
|  | Hungary | .425*** | .575*** | .573*** | .364*** | -.059 | -.330*** | 5.60 (1.13) |
|  | UK | .246*** | .469*** | .485*** | .185*** | .069 | -.232*** | 5.82 (0.97) |
| Moral Conviction | Study 1 | - | - | - | - | - | - | - |
|  | Romania | .540*** | .596*** | .571*** | .423*** | .135*** | -.208*** | 5.62 (1.14) |
|  | Hungary | .409*** | .662*** | .629*** | .432*** | -.048 | -.339*** | 5.49 (1.25) |
|  | UK | .293*** | .500*** | .462*** | .251*** | .085 | -.160*** | 5.72 (1.04) |
| Identification | Study 1 | - | .548*** | .510*** | .255*** | -.142*** | - | 5.51 (1.02) |
|  | Romania | .578*** | .606*** | .579*** | .433*** | .152*** | -.273*** | 5.53 (1.14) |
|  | Hungary | .481*** | .657*** | .661*** | .469*** | -.057 | -.323*** | 5.34 (1.18) |
|  | UK | .291*** | .464*** | .455*** | .260*** | .091* | -.175*** | 5.66 (0.95) |
| Intentions | Study 1 | - | .495*** | .444*** | .216*** | -.147*** | - | 4.74 (1.29) |
|  | Romania | .643*** | .666*** | .600*** | .511*** | .220*** | -.211*** | 4.28 (1.32) |
|  | Hungary | .439*** | .540*** | .531*** | .429*** | .059 | -.239*** | 3.76 (1.34) |
|  | UK | .382*** | .554*** | .535*** | .341*** | .214*** | -.219*** | 3.77 (1.23) |
| Action | Study 1 | - | .322*** | .312*** | .168*** | -.137*** | - | 1.09 (1.20) |
|  | Romania | .553*** | .563*** | .499*** | .409*** | .176*** | -.214*** | 3.30 (2.30) |
|  | Hungary | .394*** | .513*** | .493*** | .411*** | -.081* | -.285*** | 2.83 (2.23) |
|  | UK | .309*** | .441*** | .462*** | .303*** | .1445** | -.188*** | 3.11 (2.02) |
| Intrinsic | Study 1 | - | - | - | - | - | - | - |
|  | Romania | - | .802*** | .685*** | .599*** | .218*** | -.278*** | 5.16 (1.33) |
|  | Hungary | - | .654*** | .628*** | .661*** | .191*** | -.218*** | 4.82 (1.40) |
|  | UK | - | .526*** | .494*** | .572*** | .373*** | -.128** | 4.55 (1.28) |
| Integrated | Study 1 | - | - | .485*** | .159*** | -.219*** | - | 5.92 (1.08) |
|  | Romania | - | - | .773*** | .581*** | .150*** | -.350*** | 5.42 (1.26) |
|  | Hungary | - | - | .848*** | .600*** | .015 | -.341*** | 5.33 (1.31) |
|  | UK | - | - | .814*** | .401*** | .074 | -.333*** | 5.56 (1.10) |
| Identified | Study 1 | - | - | - | .286*** | -.075* | - | 5.62 (1.28) |
|  | Romania | - | - | - | .553*** | .092* | -.370*** | 5.75 (1.21) |
|  | Hungary | - | - | - | .569*** | -.008 | -.363*** | 5.53 (1.27) |
|  | UK | - | - | - | .404*** | .055 | -.374*** | 5.62 (1.06) |
| Introjected | Study 1 | - | - | - | - | .286*** | - | 4.17 (1.81) |
|  | Romania | - | - | - | - | .523*** | -.054 | 4.76 (1.34) |
|  | Hungary | - | - | - | - | .308*** | -.123*** | 4.43 (1.36) |
|  | UK | - | - | - | - | .612*** | .023 | 4.16 (1.31) |
| External | Study 1 | - | - | - | - | - | - | 2.00 (1.20) |
|  | Romania | - | - | - | - | - | .350*** | 3.21 (1.51) |
|  | Hungary | - | - | - | - | - | .355*** | 2.79 (1.32) |
|  | UK | - | - | - | - | - | .264*** | 3.11 (1.17) |
| Amotivation | Study 1 | - | - | - | - | - | - | - |
|  | Romania | - | - | - | - | - | - | 2.93 (1.60) |
|  | Hungary | - | - | - | - | - | - | 3.02 (1.41) |
|  | UK | - | - | - | - | - | - | 3.03 (1.26) |

*p* < .05; ***p* < .01; ****p* < .001

**References**

Agostini, M., & van Zomeren, M. (2021). Toward a comprehensive and potentially cross-cultural model of why people engage in collective action: A quantitative research synthesis of four motivations and structural constraints. *Psychological bulletin, 147*(7), 667-700. <https://doi.org/10.1037/bul0000256>

Deci, E., & Ryan, R. (1985). *Intrinsic motivation and self determination in human behavior.* Plenum.

Greenaway, K. H., Cichocka, A., van Veelen, R., Likki, T., & Branscombe, N. R. (2016). Feeling hopeful inspires support for social change. *Political Psychology, 37*(1), 89-107. <https://doi.org/10.1111/pops.12225>

Harth, N. S., Kessler, T., & Leach, C. W. (2008). Advantaged group's emotional reactions to intergroup inequality: The dynamics of pride, guilt, and sympathy. *Personality and Social Psychology Bulletin, 34*(1), 115-129. <https://doi.org/10.1177/0146167207309193>

Leach, C. W., Iyer, A., & Pedersen, A. (2006). Anger and guilt about ingroup advantage explain the willingness for political action. *Personality and Social Psychology Bulletin, 32*(9), 1232-1245. <https://doi.org/10.1177/0146167206289729>

Skitka, L. J. (2010). The psychology of moral conviction. *Social and Personality Psychology Compass, 4*(4), 267-281. <https://doi.org/10.1111/j.1751-9004.2010.00254.x>

Thomas, E. F., McGarty, C., & Mavor, K. I. (2009). Transforming “apathy into movement”: The role of prosocial emotions in motivating action for social change. *Personality and Social Psychology Review, 13*(4), 310-333. <https://doi.org/10.1177/1088868309343290>

van Zomeren, M. (2021). Toward an Integrative Perspective on Distinct Positive Emotions for Political Action: Analyzing, Comparing, Evaluating, and Synthesizing Three Theoretical Perspectives. *Political Psychology, 42*, 173-194. <https://doi.org/10.1111/pops.12795>

van Zomeren, M., Postmes, T., & Spears, R. (2008). Toward an integrative social identity model of collective action: a quantitative research synthesis of three socio-psychological perspectives. *Psychological bulletin, 134*(4), 504-535. <https://doi.org/10.1037/0033-2909.134.4.504>

Wlodarczyk, A., Basabe, N., Páez, D., & Zumeta, L. (2017). Hope and anger as mediators between collective action frames and participation in collective mobilization: The case of 15-M. *Journal of Social and Political Psychology, 5*(1). <https://doi.org/10.5964/jspp.v5i1.471>
